# Pressurized Liquid Extraction (PLE) and QuEChERS evaluation for the analysis of antibiotics in agricultural soils

**DOI:** 10.1016/j.mex.2020.101171

**Published:** 2020-12-03

**Authors:** Josiel José da Silva, Bianca Ferreira da Silva, Nelson Ramos Stradiotto, Mira Petrovic, Pablo Gago-Ferrero, Meritxell Gros

**Affiliations:** a Institute of Chemistry, São Paulo State University (UNESP), 14800-060 Araraquara, São Paulo, Brazil; b Bioenergy Research Institute (IPBEN), São Paulo State University (UNESP), 14800-060 Araraquara, São Paulo, Brazil; c Catalan Institution for Research and Advanced Studies (ICREA), Barcelona, Spain; d Catalan Institute for Water Research (ICRA), Emili Grahit 101, 17003 Girona, Spain; e University of Girona, Girona, Spain

**Keywords:** Antibiotics, Agricultural soil, Vinasse, Pressurized Liquid Extraction, QuEChERS

## Abstract

Vinasse, a liquid waste which originates from the production of ethanol fuel from sugarcane, has been widely used as soil amendment in Brazil. An important concern that arises from vinasse reuse is the dissemination of antibiotics to the environment through crop soils. This work evaluated the performance of Pressurized Liquid Extraction (PLE) and QuEChERS (quick, easy, cheap, effective, rugged and safe) to extract several multiple-class antibiotics, such as cephalosporins, fluoroquinolones, ionophores, lincosamides, macrolides, quinolones, streptogramin, sulfonamides, tetracyclines and others, from agricultural soils. The performance of several parameters was evaluated for both PLE and QuEChERS, such as the extraction temperature (for PLE), solvents composition, pH and the addition of EDTA. Both methods were able to extract most target antibiotics. However, QuEChERS showed higher recoveries for macrolides and nitroimidazoles, while PLE was more suitable for fluoroquinolones and ionophores (i.e. monensin). The use of citrate-phosphate buffer at pH 7.0, in combination with methanol for PLE and with acetonitrile for QuEChERS, provided the highest antibiotic recoveries for both methods. The use of EDTA did not increase antibiotic recovery rates for QuEChERS, while the temperature had almost no influence on the extraction efficiency in PLE.

• Citrate-phosphate buffer at pH 7.0 provided higher antibiotic recoveries for QuEChERS and PLE.
• The combination buffer-methanol provided higher recoveries for PLE.
• QuEChERS and PLE methods were able to extract most of the target antibiotics.

## Graphical abstract

**Figure:**
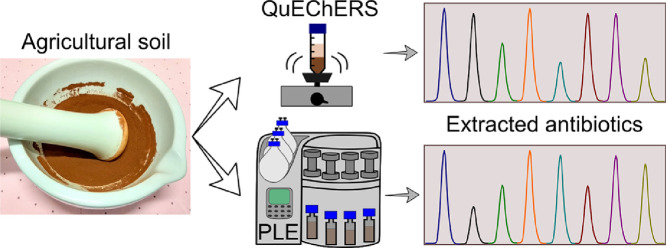

Specifications table

**Table:** 

Subject Area:	Environmental Science
More specific subject area:	Analysis of antibiotics
Method name:	Antibiotic Soil Extraction
Name and reference of original method:	Not applicable
Resource availability:	ASE 350 system (Dionex, Sunnyvale, USA) QuEChERS phase separation salts (1.5 g NaOAC and 6 g Na_2_SO_4_; 6 g Na_2_SO_4_, Agilent Technologies, Santa Clara, USA) Dispersive SPE kit Vet Drugs in Foods (50 mg PSA, 150 mg C18, 900 mg Na_2_SO_4_, Agilent Technologies, Santa Clara, USA) 5500 QTRAP hybrid quadrupole-linear ion trap tandem mass spectrometer (AB Sciex, Foster City, USA)

## Method details

### Background

The use of organic wastes for soil amendment is becoming one of the most sustainable approaches to manage the large quantity of surplus that are being generated as well as to overcome the shortage of nutrients for crop growth, especially phosphorous [1], [2], [3]. Vinasse, which originates from the production of ethanol fuel from sugarcane, has been widely used as soil amendment in sugarcane crops in Brazil. An important concern that arises from vinasse reuse is the dissemination of antibiotics to crop soils. Antibiotics are used during the ethanol production process to control the contamination by bacteria and ensure high ethanol production yields, and these compounds may persist in the soil environment once vinasse is applied as fertilizer. The analysis of organic contaminants, such as antibiotics, in soil has been performed using different traditional techniques for sample preparation, such as Soxhlet extraction, liquid partitioning with ultrasonication (USE), microwave assisted extraction (MAE) and pressurized liquid extraction (PLE). Among traditional techniques, PLE has brought several advances such as less organic solvent consumption, automation (saving analysis time) and high extraction recoveries [4]. Nevertheless, it relies on expensive instrumentation [5] not always available for all laboratories. On the other hand, QuEChERS (Quick, Easy, Cheap, Effective, Rugged and Safe) extraction has gained increased attention in the last few years within the environmental chemistry field for its higher throughput, low solvent consumption and easy to use. Furthermore, it is cheaper than PLE and thus, more available to all laboratories. QuEChERS have been successfully applied for the extraction of a wide-range of organic compounds in soil and other environmental matrices [3]. In this context, this work evaluated the efficiency of PLE and QuEChERS for the analysis of multiple class antibiotics in agricultural soils and compared the performance of both techniques. The influence of different parameters, such as extraction temperature, extraction solvents composition and pH and the use of additives on the extraction recoveries (i.e. EDTA) was evaluated. The analytical approach that provided the highest recoveries for all target antibiotics was further applied to the analysis of agricultural soils fertilized with vinasse, where the occurrence of antibiotics could be a risk.

### Reagents and standards

Chromatography grade solvents (Lichrosolv, supplied by Merck, Darmstadt, Germany), acetonitrile (ACN), methanol (MeOH) and water (HPLCw), were used for the preparation of analytical standards, samples and for target analysis by Ultra-high-performance liquid chromatography (UHPLC) coupled to quadrupole-linear ion trap (QqLIT) tandem mass spectrometry. Disodium hydrogen phosphate, trisodium citrate dihydrate, citric acid anhydrous, acetic acid (HAc) glacial HPLC grade and hydrochloric acid 1.0 mol·L^−1^ were supplied by Panreac (Barcelona, Spain). Formic acid 98% was from Merck. Sodium acetate and sodium sulfate (≥99%) were from Sigma-Aldrich (Madrid, Spain). Ethylenediaminetetraacetic acid disodium salt (Na_2_EDTA) at 0.1 mol·L^−1^ and sodium hydroxide 1.0 mol·L^−1^ solutions were from Scharlau (Barcelona, Spain). The dispersive SPE kit Vet Drugs in Foods containing 50 mg PSA (primary and secondary amine exchange material), 150 mg C18EC, 900 mg Na_2_SO_4_ was from Agilent Technologies (Santa Clara, USA). The analytical standards used for the analysis of antibiotics were purchased from Sigma-Aldrich with purity ≥ 93%. The substances used as isotopically labelled internal standards (ILIS) were purchased from Sigma-Aldrich and Toronto Research Chemicals (Ontario, Canada). Individual stock and ILIS standard solutions were prepared at a concentration of 1000 mg·L^−1^ in methanol, except quinolone and fluoroquinolone antibiotics, which were prepared in methanol with the addition of 100 µL of sodium hydroxide (NaOH) 1.0 mol·L^−1^ solution, cefalexin, which was prepared in water and ampicillin, penicillin V and ceftiofur, that were prepared in ACN:HPLCw (1:1, v/v). Furthermore, ceftiofur had a concentration of 100 mg·L^−1^. Stock solutions were used to prepare intermediate standard solutions at 20 mg·L^−1^, which in turn, were used to prepare the working standard solutions (at 500, 50 and 10 µg·L^−1^ in MeOH/HPLCw (20:80, v/v) containing all target analytes. Table 1 lists the antibiotics analyzed together with the ILIS used for their quantification.

**Table 1:** List of target analytes with the respective isotopically labelled internal standards (ILIS) used for their quantification.

Class of antibiotic	Compound	Molecular formula	ILIS
Cephalosporins	Cefalexin	C_16_H_17_N_3_O_4_S	Cefuroxime-d3
	Ceftiofur	C_19_H_17_N_5_O_7_S_3_	Ceftiofur-d3
Dihydrofolate reductase inhibitors	Trimethoprim	C_14_H_18_N_4_O_3_	Trimetoprim-d3
Fluoroquinolones	Ciprofloxacin	C_17_H_18_FN_3_O_3_	Ciprofloxacin-d8
	Enrofloxacin	C_19_H_22_FN_3_O_3_	Enrofloxacin-d5
	Marbofloxacin	C_17_H_19_FN_4_O_4_	Marbofloxacin-d8
Ionophores	Monensin	C_36_H_62_O_11_	–
	Salinomycin	C_42_H_70_O_11_	–
Lincosamides	Clindamycin	C_18_H_33_ClN_2_O_5_S	Clindamycin-d3
	Lincomycin	C_18_H_34_N_2_O_6_S	Lincomycin-d3
Macrolides	Azithromycin	C_38_H_72_N_2_O_12_	Azithromycin-d3
	Clarithromycin	C_38_H_69_NO_13_	Charithromycin-d3
	Tilmicosin	C_46_H_80_N_2_O_13_	Tilmicosin-d3
Nitroimidazole	Metronidazole	C_6_H_9_N_3_O_3_	Metronidazole-d4
	Metronidazole-OH	C_6_H_9_N_3_O_4_	Metronidazole-OH-d4
Penicillins	Amoxicillin	C_16_H_19_N_3_O_5_S	Amoxicillin-d4
	Ampicillin	C_16_H_19_N_3_O_4_S	Ampicillin-d5
	Penicillin V	C_16_H_18_N_2_O_5_S	Penicillin V-d5
Pleuromutilin	Florfenicol	C_12_H_14_Cl_2_FNO_4_S	Florfenicol-d3
	Tiamulin	C_28_H_47_NO_4_S	Tiamulin-13C4
Quinolones	Pipemidic acid	C14H17N5O3	Oxonilic acid-d5
Streptogramin	Virginiamycin M1	C_28_H_35_N_3_O_7_	Virginiamycin M1-d2
	Virginiamycin S1	C_43_H_49_N_7_O_10_	Virginiamycin M1-d2
Sulfonamides	Sulfadiazine	C_10_H_10_N_4_O_2_S	Sulfadiazine-d4
	Sulfamethazine	C_12_H_14_N_4_O_2_S	Sulfamethazine-d4
	Sulfamethoxazole	C_10_H_11_N_3_O_3_S	Sulfamethoxazole-d4
	Sulfapyridine	C_11_H_11_N_3_O_2_S	N-acetilsulfapyridine-d4
Tetracyclines	Chlortetracycline	C_22_H_23_ClN_2_O_8_	Tetracycline-d6
	Doxycycline	C_22_H_24_N_2_O_8_	Doxycycline-d3
	Oxytetracycline	C_22_H_24_N_2_O_9_	Tetracycline-d6
	Tetracycline	C_22_H_24_N_2_O_8_	Tetracycline-d6

### PLE extraction and optimization

A pool of 9 different soil samples was used for the method optimization, which was homogenized using mortar and pestle. 1.0 g of the soil pool was weighted in 20 mL amber glass vials. Samples were extracted using PLE with the Thermo Scientific™ Dionex™ ASE™ 350 system, equipped with 22 mL stainless steel extraction cells and using different extraction solvents and temperatures, as indicated in Table 2. Soil was mixed with an amount of diatomaceous earth (Dionex™ASE^Ⓡ^ Prep DE), enough to fill the extraction cells. Three glass fiber filters were placed, two in the bottom and one in top part of the extraction cells. The PLE conditions used for sample extraction were: a static time of 3 min, preheating period of 5 min, 3 extraction cycles of 5 min, flush volume of 100% and 60 s of nitrogen purge. PLE extracts were diluted with 500 mL ultrapure water and 15 mL of a 0.1 mol·L^−1^ of Na_2_EDTA solution were added. Diluted extracts were filtered through 0.45 µm PVDF filters and they were subject to SPE for extract purification using Oasis^Ⓡ^ HLB (200 mg, 6 cc) cartridges, from Waters Corporation (Milford, MA, U.S.A.). Prior to SPE, cartridges were conditioned with 5 mL HPLC grade methanol followed by 5.0 mL HPLC grade water. Sample loading was done using a flow of 3–4 drops per second. After that, the cartridges were rinsed with 5.0 mL HPLC grade water and were dried under vacuum for 25 min. The extracts were eluted with 4 × 2 mL of HPLC grade methanol, evaporated and dried under a gentle nitrogen stream, reconstituted with 500 µL of a MeOH:HPLCw (20:80, v/v) followed by its transference to the 1.5 mL amber glass vial. Next, the ILIS were added to the final extracts at a concentration of 25 µg·L^−1^.

**Table 2:** Different solvents and temperature used in the optimization of the PLE extraction method.

Test	Extraction solvent (1:1)	Temperature °C
PLE-A	Citrate buffer pH 4.0*: ACN	50
PLE-B	Citrate-phosphate buffer pH 2.6*: ACN	70
PLE-C	Citrate buffer pH 4.0*: ACN	70
PLE-D	Citrate-phosphate buffer pH 7.0*: ACN	70
PLE-E	Citrate-phosphate buffer pH 2.6*: MeOH	70
PLE-F	Citrate buffer pH 4.0*: MeOH	70
PLE-G	Citrate-phosphate buffer pH 7.0*: MeOH	70

⁎ The buffers solutions were prepared following the literature [6].

### QuEChERS extraction and optimization

Different QuEChERS methods were evaluated, as summarized in Table 3. As described for PLE studies, a pool of 9 soil samples was homogenized using mortar and pestle and 1.0 g was weighted in a 50 mL PPL centrifuge tube. After that, 5.0 mL of solvent 1 were added followed by 30 s of intense vortex shaking intended to hydrate and homogenize the sample. Subsequently, 6.0 mL of solvent 2 were added followed by 5 min of manually shaking. Next, the phase separation salt was added, and samples were vortex shaken again for 30 s. Samples were centrifuged at 10,000 rpm for 5 min at 4 °C and 4.0 mL of supernatant was transferred to a 15 mL PPL tube containing 50 mg PSA, 150 mg C18 sorbent and 900 mg of Na_2_SO_4_ followed by 30 s of vortex shaking. This mixture was centrifuged again, using the conditions described above. 3.0 mL of supernatant was collected in a glass tube and it was evaporated under a gentle nitrogen stream until dryness. The extract was reconstituted with 500 µL of a MeOH:HPLCw (20:80, v/v) mixture followed by filtration through PVDF 0.22 µm syringe filters (Merck Millipore) and the extract was transferred to the 1.5 mL amber glass vial. Next, the ILIS were added to the final extracts at a concentration of 25 µg·L^−1^.

**Table 3:** Solvents and phase separation salts used in the QuEChERS methodologies.

Method	Solvent 1	Solvent 2	Phase separation salt
Q-A	HPLC grade water	ACN with 1% HAc	1.5 g NaOAc + 6 g Na_2_SO_4_
Q-B	5.0 mL EDTA 0.1 mol·L^−1^	ACN with 1% HAc	1.5 g NaOAc + 6 g Na_2_SO_4_
Q-C	Citrate-phosphate buffer pH 2.6*	ACN	6.0 g Na_2_SO_4_
Q-D	Citrate-phosphate buffer pH 4.0*	ACN	6.0 g Na_2_SO_4_
Q-E	Citrate-phosphate buffer pH 7.0*	ACN	6.0 g Na_2_SO_4_

⁎ The buffers solutions were prepared following the literature [6].

### UHPLC-QqLIT analyses

The analysis of antibiotics was carried out by using an Ultra-High-Performance-Liquid Chromatography (UHPLC) Acquity system (Waters Corporation, MA, USA) coupled to a 5500 QTRAP hybrid quadrupole-linear ion trap tandem mass spectrometer (QqLIT ABSciex, Foster City, CA, USA), as described in detail elsewhere [7]. Briefly, the chromatographic separation was performed by using an Acquity HSS T3 column (50 mm x 2.1 mm, 1.8 µm particle size) from Waters Corporation. A gradient elution mode was applied for compound separation using two solvents, acetonitrile (A) and HPLC grade water acidified at 0.1% with formic acid (B) at a flow rate of 0.5 mL·min^−1^. The solvent program used started with 5% A and reached 70% at 3.0 min. From 3.0 to 3.5 min 100% of solvent A was used and this proportion was hold until 5.0 min. From 5.0 to 5.1 min the initial conditions were restored, and they were hold until 6.0 min for the equilibration of the column. An injection volume of 5.0 µL was used. Antibiotics were analyzed by positive electrospray ionization mode (ESI+). The mass spectrometry detection was performed using the Scheduled Multiple Reaction Monitoring (Scheduled MRM) mode, with 0.25 s of target scan time and 20 s of MRM detection window. The source ionization parameters set-up was: 30 for curtain gas, source temperature of 650 °C, 5500 V for ion spray voltage, 60 for nebulizer gas and 50 for the heater gas.

### Method validation parameters

The performance of the extraction methods was evaluated by calculating the extraction recoveries of the target antibiotics. Soil samples were spiked, with a mixture containing all antibiotics, at the concentration of 50.0 ng·g^−1^ dry weight (d.w.). Spiked samples were intensely vortexed for 30 s and they were kept for 1 h open in a laboratory fume hood to evaporate the solvent and kept closed overnight in a fridge to stabilize the interaction between the antibiotics and the soil. After that, the QuEChERS (*n* = 3) and PLE (*n* = 3) extractions tests were performed. Non-spiked samples were also analyzed to consider any background level of the target compounds. Recovery rates (*RE*) were calculated by using the equation $RE=(C_{Exp}/C_{S})*100\%$, where $C_{Exp}$ is the experimental concentration obtained after all the analytical extraction procedure by UHPLC-QqLIT analysis of the spiked samples and $C_{s}$ is the theoretical spiked concentration. The concentration of spiked samples was calculated by using the internal standard calibration approach using the ILIS, as indicated in Table 1, to correct for any potential matrix effects. After defining the most appropriate extraction method based on recovery rates, the analytical procedure was further evaluated by determining the extraction recoveries (RE) in triplicate at three different concentration levels (10.0, 30.0 and 50.0 ng·g^−1^), the matrix effects (ME), linearity, repeatability and method detection (MDLs) and quantification limits (MQLs). The matrix effect was calculated according to the literature [8] by using the equation ME=(B/A)*100%, where A is the peak area obtained for an analyte in a standard solution and B is the corresponding peak area of the analyte in a spiked extract after following the complete sample preparation protocol. The repeatability was determined by calculating the relative standard deviation (RSD) of the recovery tests at different concentrations. Method detection and quantification limits were defined as the minimum concentration to give a signal-to-noise ratio of 3 and 10, respectively, and the linearity was evaluated by linear regression without weighting in the range of 0.5–100 µg·L^−1^ (referred as concentration in the sample extracts). Calibration standards were measured at the beginning and at the end of each injection sequence, and one calibration standard was measured repeatedly every 20–25 injections, to check for signal stability.

## Results

### Extraction of antibiotics by PLE

**Fig. 1:**
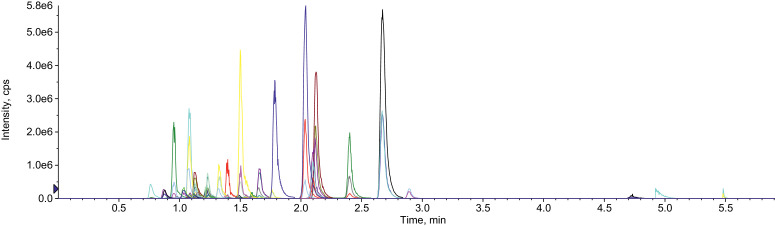
Total ion chromatogram (TIC) of a soil extract, spiked at 50 ng·g^−1^ d.w. of antibiotics, prior to PLE extraction.

Several parameters have an influence on the extraction recoveries in PLE. Among these parameters, the most critical are the extraction solvents and temperature, cycle number and time of cycle [9]. In this work, a time of cycle of 5.0 min and a number of cycles of 3 was used in all methods and were selected based on the information available in the scientific literature [4]. Different temperatures and extraction solvents were evaluated, as indicated in Table 2. High temperature can improve the solubility and diffusion rate of the analytes, but it can also contribute to the degradation of antibiotics, thus being an important factor to be evaluated. The effect of the temperature on the recovery rates of the target analytes can be observed in Table 4 (tests PLE-A and PLE-C, performed at 50 and 70 °C, respectively). Results point out that the use of a higher temperature led to an increase in the extraction efficiency for most antibiotics. Thus, a temperature of 70 °C was chosen as the optimal extraction value. Besides the temperature, the pH and the composition of the solvent extraction have an important effect on the adsorption and solubility of the analytes. The influence of the pH in the solubility of a compound is related to its ionization state, which has an influence on its capability for adsorption and thus, it directly affects the extraction. To verify the influence of the pH, tests PLE-B, PLE-C and PLE-D were performed, covering the pH values of 2.6, 4.0 and 7.0, respectively. The buffer containing the citrate ion were chosen to perform the tests because it can act as a chelating agent, preventing metallic complexes formation between antibiotics and metallic ions, favoring the antibiotic extraction [10]. Besides that, citrate-phosphate buffer has a buffering range from pH 2.6 to 7.0, [6] and permits the assessment of the effects of a broad range of pHs in the extraction efficiency. In these tests, the extraction solvent used was a mixture of buffer-acetonitrile 1:1 and the recovery obtained for each analyte is summarized in Table 4. Results obtained point out that both pH 4.0 and 7.0 favor the achievement of higher recoveries. Nevertheless, pH 7.0 was the only one that provided good recovery for salinomycin, which is an important compound in the context of this work because it is one of the antibiotics of major use in the fermentation step in ethanol production. Recovery rates using methanol instead of acetonitrile were also evaluated by using a mixture of buffer-methanol (1:1) as extraction solvent at different pH in the tests PLE-E, PLE-F and PLE-G (pH 2.6, 4.0 and 7.0, respectively). The results were similar to those obtained when using buffer-acetonitrile as a extraction solvent and pH 7.0 provided better extraction recoveries as well, as shown in Table 4. Comparing the results obtained with the two solvent combinations, the mixture buffer-methanol (PLE-G) provided better recoveries than the mixture buffer-acetonitrile, with few exceptions. For this reason, the mixture citrate-phosphate buffer pH 7.0 and methanol (1:1) was selected as the most appropriate solvent composition to perform the extraction of antibiotics in soil samples and well-defined chromatographic peaks were obtained for all target analytes, as shown in Fig. 1.

**Table 4:** Recovery rates obtained for each analyte in the different tests with PLE.

Antibiotics	%Recovery (%RSD) *n* = 3						
	PLE-A	PLE-B	PLE-C	PLE-D	PLE-E	PLE-F	PLE-G
Cefalexin	4.0 (0.4)	4.5 (3.8)	4.3 (4.5)	4.1 (2.1)	6.4 (0.9)	5.4 (5.0)	5.5 (5.6)
Ceftiofur	n.r	n.r	n.r	n.r	n.r	n.r	30.5 (9.2)
Trimethoprim	14.0 (0.7)	22.0 (2.6)	38.6 (22.0)	61.5 (2.5)	84.0 (5.4)	86.9 (3.7)	90.3 (0.5)
Ciprofloxacin	8.1 (2.1)	16.3 (3.7)	18.9 (16.3)	15.9 (18.8)	6.6 (14.3)	8.8 (0.0)	17.8 (9.1)
Enrofloxacin	32.7 (16.9)	27.8 (6.1)	22.8 (27.8)	59.8 (1.9)	3.3 (24.6)	6.5 (2.6)	41.6 (5.4)
Marbofloxacin	5.7 (10.2)	10.6 (15.7)	13.5 (10.6)	19.6 (0.8)	6.6 (35.6)	8.9 (3.3)	54.6 (5.2)
Monensin	n.r	n.r	n.r	n.r	n.r	n.r	21.2 (3.0)
Salinomycin	19.8 (21.8)	n.r	9.4 (26.8)	65.6 (8.6)	n.r	4.7 (38.5)	62.2 (9.5)
Clindamycin	21.9 (11.0)	30.1 (15.5)	45.0 (9.4)	77.2 (1.8)	76.2 (5.2)	75.0 (5.7)	90.7 (2.3)
Lincomycin	1.3 (6.5)	1.7 (5.6)	1.8 (1.7)	4.4 (5.2)	4.9 (18.4)	10.1 (3.8)	61.8 (2.3)
Azithromycin	n.r	n.r	25.4 (0.0)	7.2 (26.2)	23.3 (56.8)	n.r	19.2 (29.6)
Clarithromycin	87.3 (1.6)	82.6 (5.8)	88.3 (1.2)	77.1 (10.6)	80.6 (2.8)	80.0 (2.7)	61.6 (17.9)
Metronidazole	1.8 (7.2)	1.3 (10.0)	2.5 (11.2)	1.2 (3.4)	14.9 (0.7)	15.7 (0.9)	15.6 (0.4)
Metronidazole-OH	0.8 (1.5)	0.8 (2.7)	1.1 (7.0)	0.8 (3.1)	4.2 (0.7)	4.1 (0.7)	4.1 (1.0)
Florfenicol	90.7 (0.8)	75.9 (2.4)	79.5 (1.2)	77.3 (5.7)	74.9 (3.6)	73.5 (2.5)	76.0 (8.2)
Tiamulin	64.7 (2.8)	63.1 (5.6)	64.8 (0.9)	50.7 (2.5)	78.5 (1.6)	77.5 (3.5)	62.9 (17.8)
Pipemidic acid	10.3 (1.5)	12.0 (0.4)	12.1 (12.0)	10.8 (0.8)	13.1 (11.3)	12.6(2.2)	27.8 (2.0)
Virginiamycin M	35.5 (2.8)	65.8 (3.4)	65.0 (6.1)	55.1 (8.0)	61.9 (2.5)	62.6 (0.9)	55.3 (1.3)
Virginiamycin S	40.3 (6.0)	44.3 (1.6)	41.3 (3.8)	39.4 (1.4)	45.8 (10.5)	39.0 (9.4)	33.3 (26.8)
Sulfadiazine	14.4 (2.3)	10.6 (9.8)	18.7 (10.3)	5.8 (7.5)	44.9 (10.5)	45.4 (1.7)	50.6(3.8)
Sulfamethazine	59.2 (2.9)	48.5(2.6)	70.0 (1.6)	54.1 (6.0)	68.4 (0.8)	71.2 (14.3)	81.1 (8.2)
Sulfamethoxazole	70.0 (4.0)	78.0 (2.5)	78.7 (0.9)	45.1 (5.3)	73.6 (6.1)	70.7 (9.8)	83.5 (4.9)
Sulfapyridine	34.2 (23.2)	61.1 (18.3)	73.1 (3.3)	33.3 (3.8)	104.4 (18.2)	122.3 (1.0)	88.2 (19.9)
Chlortetracycline	24.4 (3.5)	19.4 (5.6)	23.8 (19.4)	25.8 (3.3)	16.8 (12.0)	15.8 (8.4)	20.6 (10.8)
Doxytetracycline	50.5 (3.1)	45.2 (2.5)	45.9 (17.6)	55.1 (2.8)	24.9 (1.7)	31.3 (11.3)	52.1 (0.8)
Oxytetracycline	4.1 (17.7)	4.7 (28.0)	7.7 (4.7)	n.r	16.3 (8.1)	16.3 (5.0)	11.4 (9.0)
Tetracycline	11.2 (0.5)	11.6 (12.4)	17.5 (11.6)	9.1 (0.5)	24.0 (7.1)	26.8 (8.4)	53.6 (2.1)

n.r. = non-recovered.

### Extraction of antibiotics by QuEChERS

**Fig. 2:**
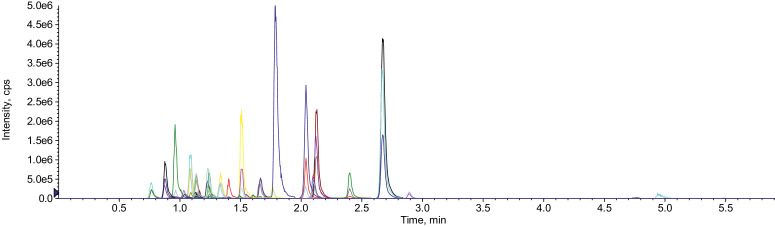
Total ion chromatogram (TIC) of a soil extract, spiked at 50 ng·g^−1^ d.w. of antibiotics, prior to QuEChERS extraction.

The efficiency of different extraction solvents and phase separation salts was investigated as described in Table 3. The test Q-A and Q-B was performed to investigate the effect of EDTA in the extraction efficiency. This reagent is a strong chelating agent and can form complexes with metal ions. Its use might help preventing the formation of complexes between metals and tetracycline, fluoroquinolone and macrolide antibiotics, improving the extraction recovery of these compounds [7]. The tests QC, QD and QE were performed using the citrate-phosphate buffer, which contains the citrate ion that can act as a chelating agent as well. Na_2_SO_4_ was used to promote the phase separation between water an acetonitrile and to dry the water phase. This salt was used instead of MgSO_4_, which is mostly used in QuEChERS protocols, because Mg^2+^ ions can also form complexes with tetracycline and fluoroquinolone antibiotics, considerably decreasing extraction recoveries [11]. The results achieved with the different conditions tested are summarized in Table 5. Comparing the results for the QA and QB tests, both buffered with acetate buffer and with extra use of EDTA in the QB test, it is possible to note that the use of EDTA did not provide an increase in the extraction efficiencies for antibiotics. The results achieved in the tests QC, QD and QE, performed with the use of citrate-phosphate buffer as an extraction solvent and at different pHs, point out that this buffer is more suitable for antibiotics extraction, since higher extraction recoveries were achieved. The use of citrate-phosphate buffer improved the extraction recoveries for tetracyclines, fluoroquinolones, cephalosporins and pipemidic acid. The influence of pH in antibiotic extraction was evidenced in the tests QC, QD and QE, performed at pH 2.6, 4.0 and 7.0, respectively. Results indicate that the buffer pH has an influence on the extraction efficiency. In general terms, the best recovery rates were achieved when working at pH 7.0 and this is especially important for lincomycin, tetracyclines, sulfonamides, salinomycin and monensin. In this way, we can conclude that the best extraction efficiencies when using QuEChERS are obtained when working under the conditions described in the QE test, Table 3, using as extraction solvent the mixture 1:1 of citrate-phosphate buffer at pH 7.0 and ACN. With these experimental conditions, well-defined chromatographic peaks were obtained for all target analytes, as shown in Fig. 2.

**Table 5:** Recoveries obtained for each analyte in the different tests using QuEChERS.

Antibiotics	%Recovery (%RSD) *n* = 3				
	QA	QB	QC	QD	QE
Cefalexin	n.r.	n.r.	9.2 (11.5)	7.7 (14.8)	13.5 (2.3)
Ceftiofur	n.r.	n.r.	41.0 (2.2)	43.6 (1.6)	39.7 (3.6)
Trimethoprim	87.6 (0.6)	84.8 (2.0)	99.8 (8.0)	99.5 (9.8)	141.7 (6.3)
Ciprofloxacin	n.r.	n.r.	13.2 (9.1)	11.9 (4.3)	5.2 (13.6)
Enrofloxacin	n.r.	n.r.	19.2 (30.3)	25.3 (25.6)	20.4 (0)
Marbofloxacin	n.r.	n.r.	17.6 (4.4)	20.5 (10.1)	10.8 (4.4)
Monensin	n.r.	n.r.	n.r.	n.r.	12.4 (0.4)
Salinomycin	40.4 (8.4)	42.3 (16.4)	n.r.	n.r.	108.7 (0.9)
Clindamycin	105.2 (0.8)	102.4 (1.1)	80.8 (8.5)	90.1 (10.9)	114.3 (3.3)
Lincomycin	22.9 (4.3)	18.1 (5.5)	49.7 (6.5)	53.9 (7.9)	82.5 (3.1)
Azithromycin	78.2 (0.7)	79.4 (5.0)	46.6 (14.7)	74.9 (10.1)	48.8 (0.5)
Clarithromycin	103.1 (7.0)	100.3 (0.4)	91.4 (4.9)	92.7 (4.5)	90.2 (1.3)
Tilmicosin	114.5 (5.3)	117 (1.9)	67.4 (6.2)	95 (5.7)	81.7 (2.3)
Metronidazole	92.2 (5.6)	97.1 (0.4)	128.3 (2.7)	134.2 (1.6)	123.8 (2.1)
Metronidazole OH	53.5 (3.4)	54.3 (1.3)	95.3 (1.5)	99.5 (0.8)	98.8 (1.7)
Florfenicol	130.0 (3.5)	130.9 (2.1)	29.5 (5.6)	36.4 (4.2)	45.8 (4.6)
Tiamulin	110.9 (3.7)	109.1 (1.7)	98.8 (2.9)	107.3 (1.6)	102.3 (1.8)
Pipemidic acid	n.r.	n.r.	28.5 (1.1)	28.4 (0.6)	27.1 (0.4)
Virginiamycin M	14.3 (22.5)	5.8 (41.7)	38.2 (1.0)	42.9 (2.9)	29.7 (6.8)
Virginiamycin S	39.5 (18.3)	26.1 (10.3)	28.2 (7.1)	31.4 (12.3)	60.8 (0.4)
Sulfadiazine	43.1 (2.3)	27.8 (14.2)	39.2 (5.5)	47.9 (7.1)	80.2 (4.4)
Sulfamethazine	73.8 (3.4)	39 (16.0)	31.6 (8.5)	39.0 (3.4)	78.8 (2.1)
Sulfamethoxazole	77.6 (7.7)	53 (6.4)	53.0 (8.1)	59.2 (3.0)	90.7 (1.6)
Sulfapyridine	53.8 (0.0)	27.7 (14.8)	30.8 (9.0)	39.0 (7.0)	109.2 (4.1)
Chlortetracycline	n.r.	n.r.	45.6 (2.8)	54.2 (4.8)	17.6 (9.1)
Doxytetracycline	n.r.	n.r.	36.7 (14.4)	50.4 (10.1)	49.3 (8.6)
Oxytetracycline	n.r.	n.r.	41.3 (16.8)	54.4 (8.7)	34.6 (7.2)
Tetracycline	n.r.	n.r.	27.7 (8.2)	34.8 (4.9)	144.7 (2.6)

n.r. = non-recovered.

### Comparison between PLE and QuEChERS

**Fig. 3:**
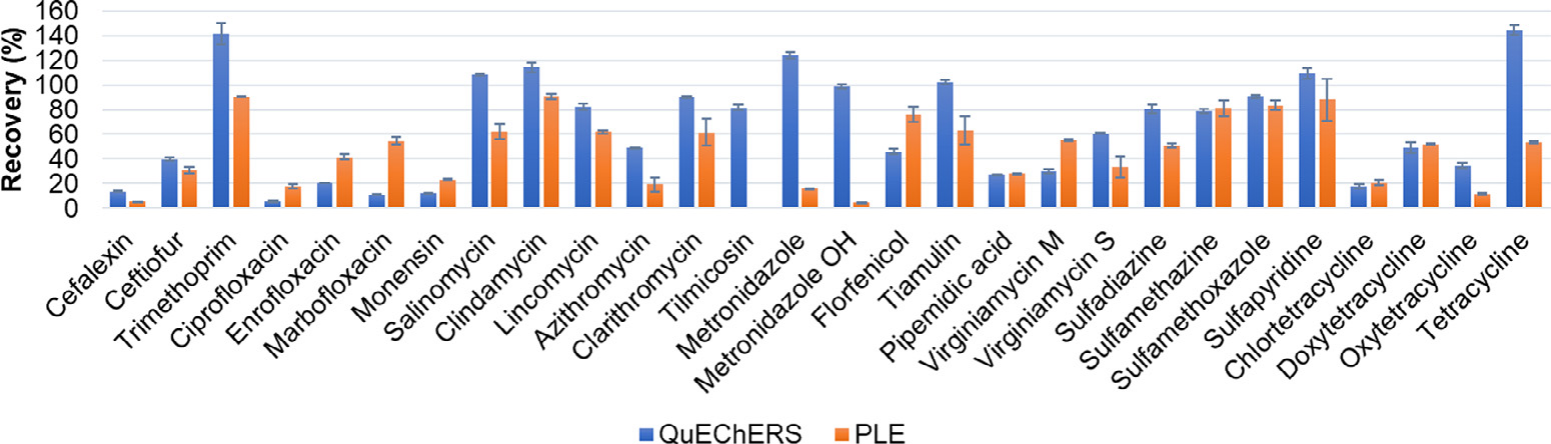
Comparison of the performance of PLE (*n* = 3) and QuEChERS (*n* = 3) in the extraction of antibiotics in soil.

The results achieved with the best extraction methods using the two methodologies tested, QuEChERS (QE test) and PLE (PLE-G test) are represented in Fig. 3 for comparison. Results highlight that both methodologies provided good recoveries for most target antibiotics, such as trimethoprim, salinomycin, lincosamides, clarithromycin, tiamulin, sulfonamides and satisfactory recoveries were obtained for fluoroquinolones, streptogramins and tetracyclines. However, penicillins and norfloxacin could not be extracted with any of the methodologies. QuEChERS was more appropriate for the analysis of the macrolides azithromycin and tilmicosin, nitroimidazoles, tetracyclines and the streptogramin virginiamycin S, while PLE is more suitable for the analysis of fluoroquinolones, the ionophore monensin, the pleuromutilin florfenicol and the streptogramin virginiamycin M. In general terms, QuEChERS provided better results than PLE, since 14 compounds showed recovery rates ≥ 60% while for PLE this was only achieved for 10 compounds. Nevertheless, the methodology choice sometimes depends on the application and the importance of each target analyte in the study to be carried out. In our case, the better extraction recovery for monensin obtained with PLE extraction, and the reasonable recoveries obtained for the other antibiotics, motivated our choice of the PLE-G method for the analysis of antibiotics in soil samples and a thorough analytical validation was performed using the conditions established in the PLE-G methodology, using the mixture 1:1 of citrate-phosphate buffer at pH 7.0 and methanol as extraction solvent. It is worth pointing out that in general, recoveries achieved by both QuEChERS and PLE methods are similar than those reported in previous studies [3,^12^,13]. Indeed, the QuEChERS and the PLE method presented here represent an improvement over some existing methods for the analysis of sulfonamides in soil [3,13]. In addition, this work targets several analytes and cover a wide range of chemical groups while other studies focus only on a limited number of compounds. Thus, the extraction efficiency of such a large array of antibiotic groups has not been addressed in previous studies, where the extraction efficiency of a limited number of compounds was evaluated. Furthermore, in previous studies, the comparison between PLE and QuEChERS was not performed [3,[12], [13], [14], [15], [16].

## Method validation

A thorough analytical validation was performed with the methodology selected for the analysis of soil samples (PLE-G method). The following parameters were calculated: recovery rates, repeatability, matrix effect (ME), linearity and limits of detection (MDLs) and quantification (MQLs). The results for the method validation parameters are summarized in Table 6. The PLE extraction method was able to provide suitable recoveries (>60%) for most target antibiotics at the spiking concentrations of 10.0, 30.0 and 50.0 ng·g^−1^ d.w. The repeatability calculated for the recovery study at 3 concentration levels, expressed as Relative Standard Deviation (RSD%), was lower than 20% for all compounds, with few exceptions. The matrix effect (ME), that could cause a signal suppression (when <100%) or signal enhancement (when >100%) ranged from 22.1 to 281.2%. These results show that target antibiotics are subject to severe matrix effects in some cases, and that a quantification approach able to correct them is needed. In this way, internal standard calibration, using isotopically labelled standards, was used for ME correction. In our study, labeled standards for almost all target compounds were available. In the case that the same exact labelled analogue could not be used, a close structurally related compound from the same chemical group was used, ensuring a good correction of ME. The linearity obtained within the concentration range of 0.5–100 µg·L^−1^ (referring to the concentration in the sample extract) was good, with r^2^ > 0.99 for all analytes. The method detection limits (MDLs) ranged from 0.03 to 3.1 ng·g^−1^ d.w. while the MQLs were between 0.1 and 10.2 ng·g^−1^ d.w. showing the capability of the method to detect antibiotics at low concentrations in soils.

**Table 6:** Method performance parameters for soil analysis.

Antibiotics	%Recovery (%RSD), *n* = 3			%ME	Linearity (r^2^)	MDLs (ng·g^−1^)	MQLs (ng·g^−1^)
	10.0 ng·g^−1^	30.0 ng·g^−1^	50.0 ng·g^−1^				
Ceftiofur	42.9 (2.8)	48.2 (3.8)	30.5 (9.2)	96.3	0.9993	0.2	0.8
Trimethoprim	128.7 (7.2)	151.1 (3.1)	138.9 (8.8)	59.4	0.9987	0.1	0.4
Enrofloxacin	35.5 (14.0)	32.9 (18.1)	26.7 (15.7)	281.2	0.9925	0.5	1.7
Marbofloxacin	34.7 (4.4)	39.2 (15.1)	38.6 (15.6)	123.7	0.9964	0.3	0.9
Monensin	22.5 (15.9)	23.2 (8.0)	21.2 (3.0)	29.1	0.9991	0.06	0.2
Salinomycin	31.4 (27.9)	55.8 (5.0)	55.1 (3.4)	82.5	0.9988	0.3	0.9
Clindamycin	127.7 (4.7)	154.4 (2.6)	152.4 (4.5)	96.8	0.9983	0.1	0.3
Lincomycin	109.0 (2.8)	120.3 (7.1)	115.5 (1.5)	74.8	0.9986	0.5	1.7
Clarithromycin	116.3 (11.7)	157.1 (4.1)	151.3 (6.9)	56.9	0.9958	0.2	0.8
Metronidazole	25.9 (16.7)	27.8 (3.6)	27.3 (9.5)	76.5	0.9976	0.2	0.8
Florfenicol	50.8 (6.1)	61.8 (10.3)	66.9 (6.8)	70.3	0.9978	0.4	1.2
Tiamulin	99.3 (9.1)	129.3 (4.0)	131.7 (9.1)	63.8	0.9982	0.06	0.2
Pipemidic acid	10.0 (0.6)	10.3 (3.5)	11.3 (3.9)	168.2	0.9868	0.7	2.4
Virginiamycin M	66.2 (2.3)	63.1 (7.2)	72.5 (2.1)	120.0	0.9970	0.03	0.1
Virginiamycin S	72.7 (3.6)	82.4 (0.6)	87.9 (0)	96.5	0.9989	0.4	1.2
Sulfadiazine	114.7 (4.8)	127.7 (14.2)	121.4 (2.9)	61.0	0.9969	0.09	0.3
Sulfamethazine	106.2 (1.8)	111.7 (1.3)	112.4 (5.4)	72.4	0.9987	0.06	0.2
Sulfamethoxazole	119.7 (2.9)	139.3 (2.1)	142.8 (4.1)	79.5	0.9976	0.06	0.2
Sulfapyridine	122.3 (19.6)	140.0 (11.5)	117.3 (8.7)	60.8	0.9957	0.1	0.5
Chlortetracycline	18.4 (6.5)	24.3 (3.6)	29.3 (26.0)	22.1	0.9983	0.7	2.5
Doxytetracycline	73.4 (4.4)	64.0 (13.1)	66.3 (19.7)	180.3	0.9962	1.4	4.8
Oxytetracycline	18.8 (10.0)	14.8 (11.3)	21.9 (38.9)	117.5	0.9935	3.1	10.2
Tetracycline	42.7 (12.3)	53.8 (8.7)	52.9 (21.8)	137.0	0.9983	2.1	7.0

## Declaration of Competing Interest

The Authors confirm that there are no conflicts of interest.

## References

[1] Sharma B., Vaish B., Monika U.K.Singh, Singh P., Singh R.P. (2019). Recycling of organic wastes in agriculture: an environmental perspective. Int. J. Environ. Res..

[2] Jiang Z.P., Li Y.R., Wei G.P., Liao Q., Su T.M., Meng Y.C., Zhang H.Y., Lu C.Y. (2012). Effect of long-term Vinasse application on physico-chemical properties of sugarcane field soils. Sugar Tech.

[3] Martínez-Piernas A.B., Plaza-Bolaños P., García-Gómez E., Fernández-Ibáñez P., Agüera A. (2018). Determination of organic microcontaminants in agricultural soils irrigated with reclaimed wastewater: target and suspect approaches. Anal. Chim. Acta.

[4] Jelić A., Petrović M., Barceló D. (2009). Multi-residue method for trace level determination of pharmaceuticals in solid samples using pressurized liquid extraction followed by liquid chromatography/quadrupole-linear ion trap mass spectrometry. Talanta.

[5] Rashid A., Nawaz S., Barker H., Ahmad I., Ashraf M. (2010). Development of a simple extraction and clean-up procedure for determination of organochlorine pesticides in soil using gas chromatography-tandem mass spectrometry. J. Chromatogr. A..

[6] G. Gomori, [16]Preparation of buffers for use in enzyme studies, in: Methods Enzymology, 1955: pp. 138–146. doi:10.1016/0076-6879(55)01020-3.

[7] Gros M., Rodríguez-Mozaz S., Barceló D. (2013). Rapid analysis of multiclass antibiotic residues and some of their metabolites in hospital, urban wastewater and river water by ultra-high-performance liquid chromatography coupled to quadrupole-linear ion trap tandem mass spectrometry. J. Chromatogr. A..

[8] Matuszewski B.K., Constanzer M.L., Chavez-Eng C.M. (2003). Strategies for the assessment of matrix effect in quantitative bioanalytical methods based on HPLC-MS/MS. Anal. Chem..

[9] Andreu V., Picó Y. (2019). Pressurized liquid extraction of organic contaminants in environmental and food samples. TrAC - Trends Anal. Chem..

[10] Moreno-González D., García-Campaña A.M. (2017). Salting-out assisted liquid–liquid extraction coupled to ultra-high performance liquid chromatography–tandem mass spectrometry for the determination of tetracycline residues in infant foods. Food Chem..

[11] Gros M., Mas-Pla J., Boy-Roura M., Geli I., Domingo F., Petrović M. (2019). Veterinary pharmaceuticals and antibiotics in manure and slurry and their fate in amended agricultural soils: findings from an experimental field site (Baix Empordà, NE Catalonia). Sci. Total Environ..

[12] Biel-Maeso M., Corada-Fernández C., Lara-Martín P.A. (2017). Determining the distribution of pharmaceutically active compounds (PhACs) in soils and sediments by pressurized hot water extraction (PHWE). Chemosphere.

[13] García-Galán M.J., Díaz-Cruz S., Barceló D. (2013). Multiresidue trace analysis of sulfonamide antibiotics and their metabolites in soils and sewage sludge by pressurized liquid extraction followed by liquid chromatography-electrospray-quadrupole linear ion trap mass spectrometry. J. Chromatogr. A..

[14] Xu M., Qian M., Zhang H., Ma J., Wang J., Wu H. (2015). Simultaneous determination of florfenicol with its metabolite based on modified quick, easy, cheap, effective, rugged, and safe sample pretreatment and evaluation of their degradation behavior in agricultural soils. J. Sep. Sci..

[15] Chitescu C.L., Oosterink E., de Jong J., Stolker A.A.M.(Linda) (2012). Ultrasonic or accelerated solvent extraction followed by U-HPLC-high mass accuracy MS for screening of pharmaceuticals and fungicides in soil and plant samples. Talanta.

[16] Popova I.E., Morra M.J., Parikh S.J. (2019). Pressurized liquid extraction of six tetracyclines from agricultural soils. J. Environ. Sci. Heal. - Part B Pestic. Food Contam. Agric. Wastes..

